# Determination of nine bioactive phenolic components usually found in apple juice by simultaneous UPLC‐MS/MS

**DOI:** 10.1002/fsn3.3399

**Published:** 2023-05-04

**Authors:** Qiu‐lin Li, Fu‐xin Chen, Zi‐teng Luo, Meng‐rang Wang, Xiang Han, Jun‐feng Zhu, Juan E. Li, Jing Liu, Kan‐she Li, Pin Gong

**Affiliations:** ^1^ School of Chemistry and Chemical Engineering Xi'an University of Science and Technology Xi'an China; ^2^ School of Food Science and Engineering Shaanxi University of Science and Technology Xi'an China; ^3^ Shaanxi Provincial People's Hospital Xi'an China

**Keywords:** active components, apple juice, phenolic components, quality control, UPLC‐MS/MS

## Abstract

The functional food ingredients of apple juice can significantly change during processing, transportation, and storage, thus affecting the quality of the product. A simple and derivation‐free analytical method based on ultra‐high‐performance liquid chromatography–tandem mass spectrometry (UPLC‐MS/MS) was developed and optimized for the simultaneous determination of functional food ingredients in apple juice bought in the market. Cleanup steps and chromatographic conditions were optimized to remove interference and decrease the matrix effect. The nine target analytes were separated on an Acquity UPLC system equipped with a BEH C18 column and detected by electrospray ionization source (ESI) operating in positive subsection acquisition mode under multiple reaction monitoring (MRM) conditions. The results showed that p‐hydroxybenzoic acid, protocatechuate, caffeic acid, chlorogenic acid, epicatechin, phloridzin, hyperoside, procyanidin B_2_, and rutin could be sufficiently separated for content determination within 6 min. In the concentration range of 20 μg/L–50 mg/L, nine standard samples exhibited a good linear fit with correlation coefficients above .985.

## Graphical abstract

In this paper, nine phenolic active small molecules of apple products were determined by ultra‐high‐performance liquid chromatography and electrospray ionization tandem mass spectrometry. By utilizing the advantages of UPLC‐MS/MS separation in MRM mode, we developed a protocol that is easy to implement, with a total analysis time required for chromatographic runs (including sample injection and column equilibration) of only 6 min. The standard curves of compounds had an *R* ^2^ > .98, and the protocol was used for the analysis of complex food samples.

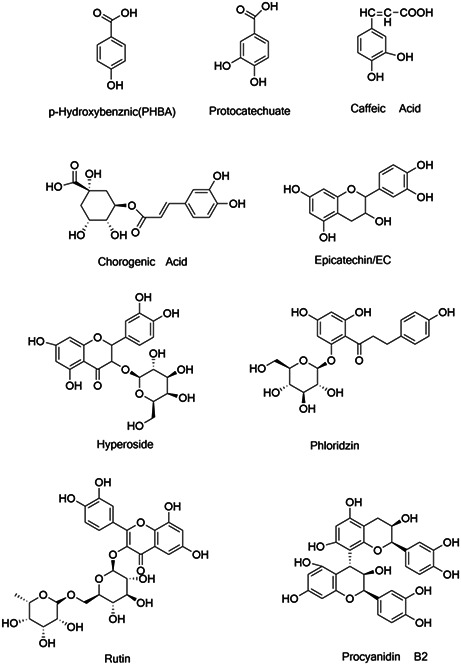

## 1 INTRODUCTION

“An apple a day keeps the doctor away” is a common parental advice, and the apple is one of the most widely consumed fruits worldwide (Serra et al., 2010). Apples are a source of various biologically active substances, especially phenolic compounds (Chen et al., 2021; Wu et al., 2007). Many studies show that phenolics have antifungal, antimicrobial, antioxidant, anticancer, antidiabetic, and antihypertensive properties, while also reducing the risk of cardiovascular diseases and obesity (Alu'datt et al., 2017; Tian et al., 2017). In commercial apple varieties (Malus Domestica), chlorogenic acid, catechin, epicatechin, phloretin glycosides, procyanidin B_2_, and quercetin glycosides are the main effective antioxidant phenolics (Jakobek et al., 2013; Lee et al., 2003). However, the functional food ingredients of apple juice can significantly change during processing, transportation, and storage, thus affecting the final product quality.

Chlorogenic acid is an important biologically active dietary polyphenol with various important therapeutic effects such as antioxidant, antibacterial, hepatoprotective, cardioprotective, and anti‐inflammatory activities. In addition, chlorogenic acid was found to positively influence the metabolism of lipids and glucose in hereditary and acquired metabolic disorders. It was shown that chlorogenic acid is a natural and safe food additive that can be used in place of synthetic chemical growth promoters (antibiotics) and immunopotentiators (Naveed et al., 2018). Evidence from studies in humans and experimental animals suggested that consumption of epicatechin (EC) and EC‐rich foods may improve insulin sensitivity. Cremonini et al. (2016) investigated the ability of EC supplements to prevent insulin resistance in high‐fat diet (HFD)‐fed mice. The results indicate that eating EC‐rich foods may be a viable dietary strategy to reduce obesity‐related insulin resistance (Cremonini et al., 2016). Phlorizin is a natural product and a dietary constituent found in a number of fruits that have already been approved as a food additive for long‐term use with few side effects. Phlorizin was found to activate the Tyk2/STAT3 signaling pathway to induce thermogenesis in brown adipose tissue, with therapeutic potential for the treatment of obesity and comorbidities (Yuan et al., 2016). Proanthocyanidin B2 is a highly effective natural antioxidant that can remove free radicals and inhibit the occurrence of lipid peroxidation, helping maintain the homeostasis between free radicals and antioxidant enzymes in the body and prevent related diseases (Acosta‐Estrada et al., 2014).

In addition, high concentrations of protocatechuate in apple peels have been shown to exert anti‐inflammatory, antihyperglycemic, and antiapoptotic activities in animal studies using rats and mice (Yoswaris et al., 2015). Apples also contain hyperoside, which can alleviate high glucose‐induced vascular inflammation (Tian et al., 2018). Caffeic acid can attenuate lipopolysaccharide‐induced disease behavior and neuroinflammation in mice (Mallik et al., 2016). Rutin is important for the aroma of certain apple cultivars (Damin et al., 2019) and p‐hydroxybenzoic acid (PHBA) is a controversial class of chemicals used as cosmetic and pharmaceutical preservatives (Guo et al., 2014). However, some experimental evidence suggests that it is also an endocrine disruptor and that there is an association between human exposure to parabens and adverse health outcomes (Kang et al., 2002).

Fruit juice could be a great alternative to whole fruits (Wootton‐Beard & Ryan, 2011). Compared with fruits, fruit juice has the advantages of convenient carrying, long shelf life, and few seasonal restrictions. Its fluidity makes it easier for the elderly and children to ingest. Juice demand is growing rapidly as consumers are looking for convenient fruit products with good taste and nutritional qualities (Teck‐Chai et al., 2012).

Apple juice is one of the most popular fruit juices in the world because of its agreeable taste, easy processing leading to a low price, and widely known health benefits (Wodarska et al., 2016). Currently, apple juice is consumed by millions of people every day (Tian et al., 2018). Polyphenolic compounds account for most of the antioxidant activity of apples and are distributed in the skin, pulp, and seeds. However, most of these apple polyphenols are removed during fruit juice production (Pei et al., 2017). Clarification is the step in which most antioxidants are lost from the final product, significantly reducing the polyphenol content because these compounds are mainly found in the pulp (Fahad et al., 2018). Phenolic compounds are sensitive to process conditions during juice extraction as well as further thermal and nonthermal processing (Kong et al., 2016). In order to determine whether the phenolic small molecules in apples are preserved in apple juice, the nine active small molecules reported above were selected to study their contents in various apple drinks (Matos et al., 2017).

Finding an efficient and reliable analytical method to simultaneously measure these nine compounds is a challenge. So far, several analytical methods have been reported for the determination of phenolic substances in food samples. These methods include flow injection methods, the Fast Blue BB method, GC–MS, and LC–MS methods. Costin et al. (2003) used the Fast Blue BB method to estimate total phenol/antioxidant levels in wine, and although the method provided the total phenolic content quickly, it could not quantify a specific compound (Costin et al., 2003). Medina (2011) quantified polyphenols or phenols by direct interaction with Fast Blue BB in an alkaline medium. This method is economical but is also not specific and can only detect the total content of a class of substances (Medina, 2011). Zuo et al. (2002) used GC–MS to detect phenolic substances in food samples (Zuo et al., 2002). However, GC‐based methods are not suitable for the determination of nonvolatile or thermally labile substances. Some highly sensitive methods were also used for the analysis of functional food ingredients such as HPLC‐FLD (Bi et al., 2018). In order to facilitate the analysis of phenolic compounds, it is necessary to avoid derivatization, as this takes a significant amount of time. As an important modern method, LC–MS is highly favored in the food industry due to its simplicity, speed, sensitivity, and accuracy (Bi et al., 2018). In this study, we applied the advantages of this method to establish a rapid protocol for the determination of nine representative phenolic active substances in apple juice.

## 2 MATERIALS AND METHODS

### 2.1 Sample source

In this experiment, 18 major brands of apple juice commonly found in the Chinese market were selected. Among them are seven kinds of pure apple juice (PAJ1‐7), all of which are labeled as 100% pure apple juice, with the ingredient list indicating 100% apple, or containing only water and apple juice concentrate. Six medium‐sized composite apple drinks (MAJ1‐6) have a more complex ingredient list with more ingredients, but they contain apple juice concentrate, most of which are in the fourth or fifth place. Three apple‐derived apple vinegar drinks (VAJ1‐3) contain basically apple vinegar concentrate and apple juice concentrate in the ingredient list. In one apple‐flavored carbonated beverage and one apple‐flavored lactic acid bacteria beverage (OAJ1‐2), the ingredient list contains apple juice concentrate, but is ranked lower. A representative sample consisting of at least three samples collected at different retail stores or large supermarkets for a period of 3 months (January to March 2021).

### 2.2 Materials and standards

**FIGURE 1:**
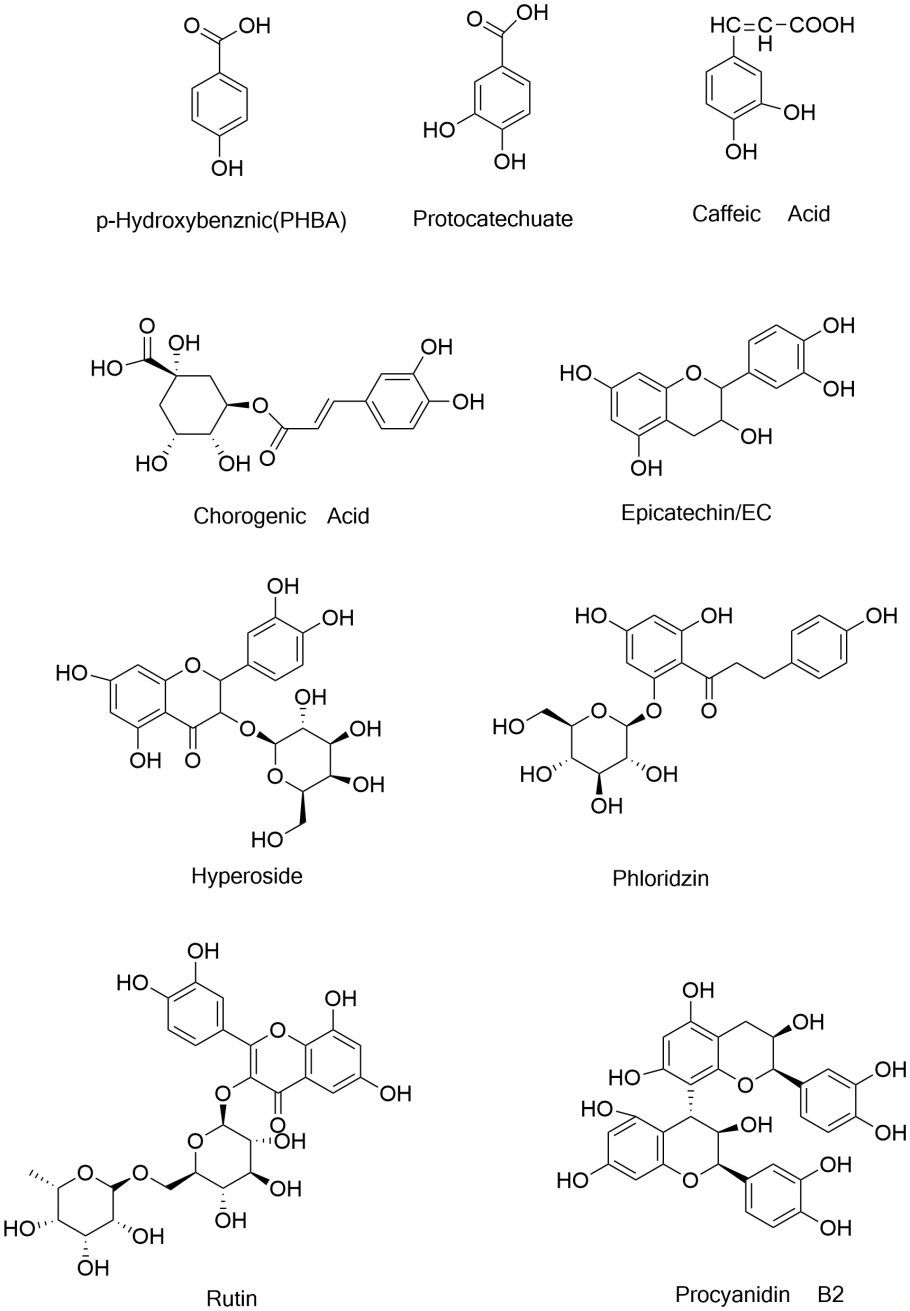
Nine phenolic active molecular compound structures.

Reference standard for nine phenolic active small molecules, i.e., hyperoside, epicatechin, caffeic acid, phloridzin, protocatechuate, procyanidin B_2_, chlorogenic acid, rutin, PHBA sourced from Shanghai Biological Technology Co., Ltd. with the purity of HPLC grade 98%–99%. The structure of the compound is shown in Figure 1. Methanol and acetonitrile were of HPLC grade from American Tedia company and water for ultrapure water.

ACQUITY system ultra‐high‐performance liquid chromatography–tandem quadrupole mass spectrometry (Waters, companies in the United States, including automatic dual gradient pump, injector, column temperature box, and Masslynx4.1 mass Spectrometry workstation).

### 2.3 Preparation of the test solution

Preparation of apple extract solution: 2‐mg apple extract dissolved in 1 mL of acetonitrile, mixed the solution at a centrifugal speed of 1280 *g*, 600‐μL supernatant was taken and dried, 1 mL of acetonitrile and 200‐μL methanol added in 600‐μL supernatant.

Preparation of apple juice solution: 400‐μL apple juice was taken in the pipette and added to 600‐μL acetonitrile solution, mixed the solution at a centrifugal speed of 1600 rpm, 600‐μL supernatant was taken and dried, 1 mL of acetonitrile and 200‐μL methanol added in 600‐μL supernatant.

### 2.4 The preparation of standard solutions

The PHBA, protocatechunate, caffeic acid, chlorogenic acid, epicatechin, phloridzin, hyperoside, procyanidin B_2_, and rutin standard were weighed in a certain mass using a high‐precision analytical balance. The hydroxybenzoic acid standard was prepared as 20, 10, 5, 2, 1, 0.2, and 0.02 μg/mL in a 7:3 ratio of methanol and water as solvents, respectively.

### 2.5 Chromatographic and mass spectrometric conditions

Chromatographic column: WATERS ACQUITY UPLC BEH C18 (2.1 mm × 50 mm, 1.7 μm) column; The flow rate of 0.2 mL/min; Column temperature: 40°C; Sample room temperature: 10°C; The sample size is 5 μL; Flow phase: 0.1% formic acid/5% acetonitrile (A)–0.1% formic acid/acetonitrile (B); The gradient elution procedure is as follows: 0–1 min, 100% (A); 1–4 min, 100%–1%; 4–5 min, 1% (A); 5–5.5 min, 1%–100% (A); 5.5–6 min, 100%.

Mass spectrum condition: using electrospray ionization source (ESI), capillary ionization voltage 3 kV, ion source temperature 150°C; solvent gas N_2_, velocity 800 L/h, solvent temperature 350°C; collision gas Ar. The multi‐reactive ion monitoring (MRM) method was used and the results are shown in Table 1. The same test conditions are applied to all juice products purchased in the market.

**TABLE 1:** Condition parameters of mass spectrum analysis.

Compound	Detection window (min)	Parent ion^c^ [M–H]^−^ (m/z)	Daughter ion (m/z)	Cone voltage (V)	Collision energy (eV)
Chlorogenic acid	2.96	353.23	191.35	35	20
PHBA	3.07	137.19	93.25	35	20
Procyanidin B_2_	3.03	577.08	425.2	36	16
Epicatechin/EC	3.21	357.19	289.19	42	12
Caffeic acid	3.11	179.13	134.27	40	20
Rutin	3.31	677.2	609.39	40	20
Hyperoside	3.42	463.3	300.19	52	26
Protocatechuate	3.65	153.13	109.11	35	20
Phloridzin	3.84	435.34	273.2	35	20

## 3 RESULTS AND DISCUSSION

### 3.1 Optimization of UPLC‐MS/MS conditions

The obtained multiple reaction monitoring (MRM) transition parameters of UPLC‐MS/MS for the determination of the nine phenolic compounds are summarized in Table 1. MS spectra were studied in both positive and negative ion modes. In the negative ion mode, the response value was higher, as was the sensitivity for some of the compounds, and the mass spectrum obtained in the negative ion mode was clearer, so that the separation of each ion peak was more obvious and easier to recognize. Therefore, the negative MS ion mode was chosen for further study.

The precursor–product ion pairs for MRM detection were generated by the Intellistart protocol (automatic tuning and calibration of the Waters Xevo TQD), which was embedded in the MassLynx software. Otherwise, the signal of each compound has to be manually optimized by altering the cone voltage and collision energy.

### 3.2 Results of separation

**FIGURE 2:**
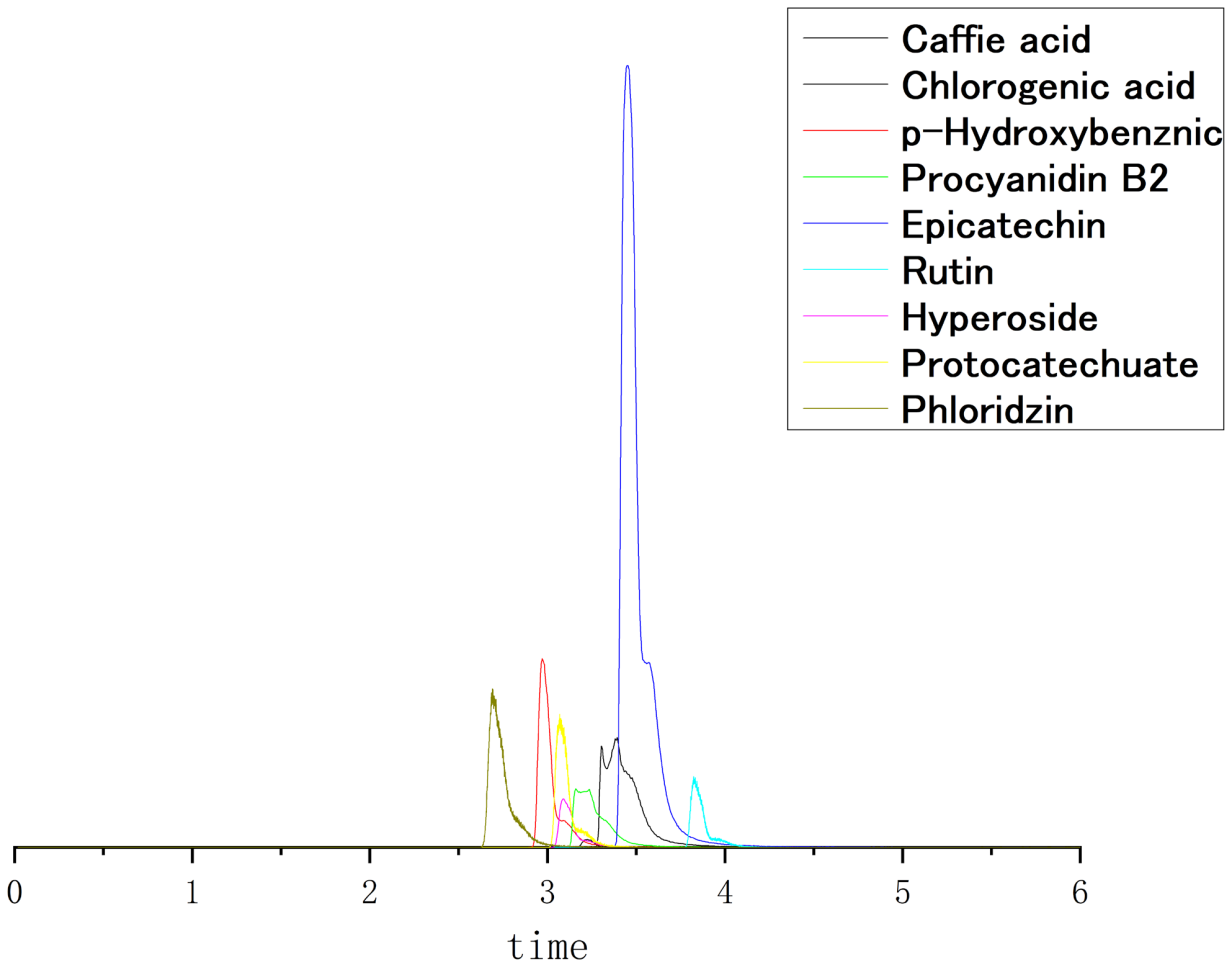
A total ion chromatogram of a standard solution mixture (50 μg/mL).

Under the above conditions, the nine apple phenolics exhibited a good separation degree within 6 min at the same run time. If only for separation, the time can be shorter (up to 3 min in this study). However, in order to better maintain reproducibility and protect the chromatographic column from the accumulation of impurities in actual biological samples. We should better extend the retention time to 6 min. A sample comprising 50 μg/mL of an authentic reference standard was used to optimize single extraction, and the total ion chromatograms of the mixture are shown in Figure 2, respectively.

### 3.3 Linear range

Using the above method for the preparation of standard solutions, the integral of the peak area was plotted as the ordinate Y, the concentration as the abscissa X. The weight coefficient was 1/X, and the standard curve was obtained by fitting to a linear equation (Table 2). The linear range of compound quantification was obtained by diluting each stock solution with a mixed solution of methanol and water to obtain seven different concentrations. The correlation coefficients were between .98574 and .99797.

**TABLE 2:** Calibration curve, correlation coefficient of nine compounds.

Compounds	Regression equation	Correlation coefficients
p‐hydroxybenzoic	*y* = 3187.0808*x* + 329.9000	.98587
Protocatechuate	*y* = 504.8337*x* + 20.3409	.98574
Caffeic acid	*y* = 228.7363*x* − 770.31058	.98763
Chlorogenic acid	*y* = 2342.4055*x* + 59.45807	.99595
Epicatechin	*y* = 888.9571*x* + 491.9744	.99797
Phloridzin	*y* = 4688.0067*x* + 84.76918	.99445
Hyperoside	*y* = 819.7575*x* − 31.70137	.99568
Procyanidin B_2_	*y* = 1013.6003*x* − 48.7589	.98887
Rutin	*y* = 469.86771*x* + 130.2538	.98953

### 3.4 Determination of functional food ingredients

The 18 batches of samples prepared using the above method were measured and the contents of each component in the samples were calculated, as shown in Table 3.

**TABLE 3:** The content of nine compounds in 18 samples.

Number	Compounds (mg/L)								
	PHBA	Protocatechuate	Caffeic acid	Chlorogenic acid	Epicatechin	Phloridzin	Hyperoside	Procyanidin B2	Rutin
PAJ1	ND	ND	ND	0.06	ND	0.0447	ND	ND	ND
PAJ2	ND	ND	ND	0.1193	ND	0.012	ND	ND	ND
PAJ3	ND	ND	ND	0.0451	ND	0.0544	ND	ND	ND
PAJ4	ND	ND	ND	0.036	ND	0.1399	ND	ND	ND
PAJ5	ND	ND	ND	ND	ND	0.0315	ND	ND	ND
PAJ6	ND	ND	ND	ND	ND	ND	ND	ND	ND
PAJ7	ND	ND	ND	ND	ND	ND	ND	ND	ND
MAJ1	ND	ND	ND	0.0599	ND	0.0436	ND	ND	ND
MAJ2	ND	ND	ND	0.0782	ND	0.0591	ND	ND	ND
MAJ3	ND	ND	ND	ND	ND	0.0339	ND	ND	ND
MAJ4	ND	ND	ND	2.6046	ND	0.2206	0.2689	ND	ND
MAJ5	ND	ND	ND	0.0876	ND	0.0341	0.1435	ND	ND
MAJ6	ND	ND	ND	ND	ND	0.0347	ND	ND	ND
VAJ1	ND	ND	ND	0.034	ND	0.0439	ND	ND	ND
VAJ2	ND	0.278	ND	0.0446	ND	ND	ND	ND	ND
VAJ3	ND	ND	ND	0.0486	ND	ND	1.1957	ND	ND
OAJ1	ND	ND	ND	ND	ND	0.0347	ND	ND	ND
OAJ2	ND	ND	ND	ND	ND	ND	ND	ND	ND

ND indicates not detected.

Protocatechuate was only detected in one sample, while chlorogenic acid was detected in 11, phloridzin in 13, and hyperoside in three of the 18 samples. The contents of PHBA, caffeic acid, epicatechin, proanthocyanidin B2, and rutin were below the detection limit. All apple vinegar samples contained chlorogenic acid at concentrations of 0.03–0.05 mg/L.

The detection and analysis method established in this article has significantly higher sensitivity compared to other research and analysis methods such as HPLC (Alu'datt et al., 2017), GC–MS (Zuo et al., 2002), and The Fast Blue BB method (Medina, 2011). Moreover, this characteristic of being able to simultaneously determine nine phenolic compounds in this article is significantly more efficient. Some studies have shown that most polyphenols in apple juice are not heat resistant, and heat treatment may lead to degradation or hydrolysis (Tian et al., 2018). Therefore, different processing techniques for apple juice may lead to the loss of phenolic substances in apple juice to varying degrees. In addition, different apple varieties selected for commercial fruit juices also have different effects on the determination of phenolic compounds. Therefore, further research involving more factors is interesting.

The compounds were detected but their distribution was not specific, and the content was not necessarily higher than in other types of beverages. Therefore, these findings cannot be used as a basis for judging that certain products contain more phenolic substances than other types of apple juice. The vast majority of consumers prefer juices that are more cloudy, higher in phenolic compounds, and considered more natural (Wodarska et al., 2016). But the type of juice is not the main factor determining the content of phenolics. The loss of phenolic substances in marketed apple juice compared to the whole fruit before processing is very significant.

## 4 CONCLUSION

By utilizing the advantages of UPLC‐MS/MS separation in MRM mode, we developed a protocol that is easy to implement, with a total analysis time required for chromatographic runs (including sample injection and column equilibration) of only 6 min. The results meet the testing requirements for the determination of material content in apple products. At the same time, the method can also be used to detect ingredients in other fruits and vegetables. The results of this study indicate that phenolic compounds were not effectively retained in the 18 tested commercially available apple beverage products. Notably, the type of juice does not appear to be directly related to the amount of phenolics. Nevertheless, there are many juices that can add extra nutrients, which may be added to compensate the shortcomings of the commercial apple juice processing.

## AUTHOR CONTRIBUTIONS

**Qiulin Li:** Methodology (equal); visualization (equal); writing – original draft (equal); writing – review and editing (equal). **Fuxin Chen:** Conceptualization (equal); funding acquisition (equal); writing – original draft (equal); writing – review and editing (equal). **Ziteng Luo:** Formal analysis (equal); investigation (equal). **Mengrang Wang:** Data curation (equal); formal analysis (equal); investigation (equal). **Xiang Han:** Resources (equal); software (equal). **Junfeng Zhu:** Investigation (equal); software (equal). **JuanE Li:** Supervision (equal); validation (equal). **Jing Liu:** Investigation (equal); supervision (equal). **Kanshe Li:** Resources (equal); supervision (equal). **Ping Gong:** Conceptualization (equal); data curation (equal); project administration (equal); supervision (equal).

## CONFLICT OF INTEREST STATEMENT

The authors declare that there is no conflict of interest and that they do not have any possible conflicts of interest.

## Data Availability

The data that support the findings of this study are available from the corresponding author upon reasonable request.
